# The C3aR promotes macrophage infiltration and regulates ANCA production but does not affect glomerular injury in experimental anti-myeloperoxidase glomerulonephritis

**DOI:** 10.1371/journal.pone.0190655

**Published:** 2018-01-09

**Authors:** Jonathan Dick, Poh-Yi Gan, A. Richard Kitching, Stephen R. Holdsworth

**Affiliations:** 1 Centre for Inflammatory Diseases, Monash University Department of Medicine, Clayton, Victoria, Australia; 2 Department of Nephrology, Monash Health, Clayton, Victoria, Australia; 3 Department of Paediatric Nephrology, Monash Children’s Hospital, Monash Health, Clayton, Victoria, Australia; Instituto Nacional de Ciencias Medicas y Nutricion Salvador Zubiran, MEXICO

## Abstract

The anti-neutrophil cytoplasmic antibody (ANCA) associated vasculitides are autoimmune diseases associated with significant morbidity and mortality. They often affect the kidney causing rapidly progressive glomerulonephritis. While signalling by complement anaphylatoxin C5a though the C5a receptor is important in this disease, the role of the anaphylatoxin C3a signalling via the C3a receptor (C3aR) is not known. Using two different murine models of anti-myeloperoxidase (MPO) glomerulonephritis, one mediated by passive transfer of anti-MPO antibodies, the other by cell-mediated immunity, we found that the C3aR did not alter histological disease severity. However, it promoted macrophage recruitment to the inflamed glomerulus and inhibited the generation of MPO-ANCA whilst not influencing T cell autoimmunity. Thus, whilst the C3aR modulates some elements of disease pathogenesis, overall it is not critical in effector responses and glomerular injury caused by autoimmunity to MPO.

## Introduction

The anti-neutrophil cytoplasmic antibody (ANCA) associated vasculitides (AAV) are diseases in which autoimmunity to the neutrophil granule proteins myeloperoxidase (MPO) or proteinase-3 (Pr3) can cause multi-organ injury, including rapidly progressive glomerulonephritis. The pathogenesis of AAV involves multiple steps. T and B cell tolerance to MPO or Pr3 is lost, resulting in the secretion of autoantibodies (ANCA). ANCA can bind to their cognate autoantigen on primed neutrophils, inducing them to activate[1] and lodge in the glomerulus. These intraglomerular neutrophils degranulate, producing reactive oxygen species and causing direct glomerular injury. Degranulation results in extensive glomerular deposits of non-leukocyte associated MPO in patients with AAV [2]. Murine models suggest that MPO-specific effector T cells recognize MPO deposited in glomeruli and contribute to glomerular injury [3,4].

The complement system is an important component of innate immunity. Three pathways can activate complement: the classical, alternative and lectin. These pathways converge on the generation of a C3 convertase. C3a is a bioactive split product of C3 produced, along with C3b, by the action of the C3 convertases. C3a is rapidly inactivated by cleavage of the C-terminal arginine to form C3a-desArg. The cellular receptor for C3a, the C3aR, is a G-protein coupled receptor with 7 trans-membrane domains and high homology to the human C5aR1. Activation of the receptor leads to intracellular calcium mobilisation [5,6].

Complement has emerged as an important mediator of disease in AAV. Murine studies revealed that complement, activated via the alternative pathway and signalling through C5aR is required for ANCA-induced neutrophil activation and glomerulonephritis [7–10]. Supporting evidence from human cohorts include elevated circulating complement activation products in active disease [11], the association of low serum C3 levels with adverse outcomes [12,13], and evidence of complement deposition in biopsies of patients with AAV [14,15]. The proof of concept phase II CLEAR study showed that the small molecule C5aR inhibitor CCX168 (Avacopan) was non-inferior to glucocorticoids for induction therapy in AAV [16]. This strategy is currently the subject of a phase 3 clinical trial (NCT02994297) in acute AAV.

Although circulating levels of C3a are elevated in patients with active AAV [11], whether C3a is pathogenic in this disease is not known. The only relevant published work to date in AAV has been the finding that C3a does not prime isolated neutrophils for activation by ANCA *in vitro* [8]. This is consistent with the inability of C3a to cause chemotaxis or degranulation in neutrophils[17]. However, AAV is a disease with the complex participation of multiple innate and adaptive immune components. Thus, as signalling through C3aR has been implicated in several relevant processes, including neutrophil mobilisation [18], the generation of T cell [19] and B cell [20]responses, macrophage recruitment [21] and mast cell degranulation [6] there are multiple potential mechanisms by which the C3aR may participate in AAV. We therefore examined the role of signalling through the C3aR in anti-MPO autoimmunity and renal injury, by studying *C*3ar^-/-^ mice[22] in two complementary models of anti-MPO glomerulonephritis.

## Materials and methods

All mice were on a C57BL/6 background. C3ar^-/-^ mice [22] were kindly provided by Professor Rick Wetzel, University of Texas. Mice were bred and housed in specific pathogen free conditions (Monash Medical Centre Animal Facility, Clayton, Victoria, Australia). Studies were performed in accordance with the National Health and Medical Research Council’s Australian code for the care and use of animals for scientific purposes and were approved by Monash University Animal Ethics committee (MMCB12/33). Mice had free access to food and water and were monitored daily by both researchers and animal facility staff. Mice were humanely euthanized with CO_2_ at the end of experiments.

### Genotyping

DNA was isolated from mouse tail clippings by ispropanol precipitation. The isolated DNA was used as a template for PCR with 0.5μM of each of the primers C1 (TACAATATAGTCAGTTGGAAGTCAGCC), NeoA (TGGGCTCTATGGCTTCTGAGGCGGAAAG), and A201+ (GAGAATCAGGTGAGCCAAGGAGAAG). GoTaq Green Master Mix (Promega) was used for the PCR reaction. The PCR was run at 95°C for 1 minute, followed by 40 cycles of 95°C for 15sec, 57°C for 15sec, 72°C for 30sec, then a final elongation step at 72°C for 30sec and a holding step of 4°C. The primers C1 and NeoA yield a fragment of 537 bp denoting the C3ar^-/-^ allele. Primers C1 and A201+ yield a fragment of 726 bp, denoting WT allele. All mice tested had the C3ar^-/-^ genotype (S1 Fig).

### Induction of glomerulonephritis and assessment of autoimmunity

Anti-MPO IgG was generated by immunising Mpo^-/-^ mice with native murine MPO generated as previously described[23]. Briefly mice were immunised sub-cutaneously (s.c.) with 15μg MPO in FCA (Sigma-Aldrich, St Louis, MO) on day 0 followed by 10μg in Freund’s incomplete adjuvant on days 7 and 14. On day 24 mice were humanely killed and blood was obtained. IgG fractions were obtained by Protein G affinity chromatography (GE Healthcare) and then extensively dialysed against PBS.

To induce glomerulonephritis age matched 6–10 week old C57B/L6 mice and C3ar^-/-^ mice received 100μg/g body weight anti-MPO IgG by tail vein injection. 1 hour later 0.5 μg/g bacterial lipopolysaccharide (LPS) (Sigma-Aldrich) was administered by a single intra-peritoneal injection. After 6 days, 24 hours before the end of experiments, mice were placed in metabolic cages for urine collection.

Autoimmune anti-MPO glomerulonephritis mice was induced as previously described [3]. Briefly, mice were immunised s.c. with 20μg MPO in FCA followed by 10μg MPO in FIA on day 7. On day 16 glomerulonephritis was triggered by injection of 0.12mg/g sheep anti-mouse GBM globulin in two divided doses. Mice were placed in metabolic cages on day 20 for assessment of proteinuria before being euthanased on day 21 by CO_2_ inhalation.

### Assessment of renal injury

Glomerular abnormalities were assessed on periodic acid-Schiff (PAS) stained, 4μm, formalin fixed, paraffin embedded sections using coded slides. Abnormalities scored included mesangial proliferation, capillary wall thickening, glomerular necrosis (defined as accumulation of PAS positive material combined with hypocellularity) and crescent formation (defined as two or more cells visible in Bowman’s space). A minimum of 30 glomeruli per mouse were examined. To evaluate leukocyte infiltrate, kidneys were fixed in periodate lysine paraformaldehyde for four hours then washed with 20% sucrose. 5μm sections were stained with a three layer immunoperoxidase technique [24] using GK1.5 (anti-CD4), FA11 (anti-CD68) or Gr1 (anti Ly6g/c). The secondary antibody was rabbit anti-rat biotin (DAKO). A minimum of 30 glomeruli per section were scored. Albuminuria was measured by ELISA (Bethyl. Montgomery, TX). Creatinine was measured using standard methods at the biochemistry laboratory, Monash Medical Centre.

### Assessment of immunity

Immunity was assessed on day 10 after immunisation with 20μg MPO in FCA and at day 21, at the end of the autoimmune anti-MPO glomerulonephritis model. The spleen and draining lymph nodes were harvested and a single cell suspension was obtained. IFN-γ and IL-17A ELISPOT was performed according to the manufacturer’s instructions (Ebioscience, San Diego, CA) with 5x10^5^ cells per well. Cells were incubated for 18 hours at 37°C with 10μg/ml heat inactivated recombinant murine MPO (rMPO). Total anti-MPO IgG was measured by ELISA on MPO coated plates, with IgG detected with sheep-anti mouse IgG-HRP (Sigma-Aldrich). Antibody subclasses were measured using subclass specific goat anti-mouse IgG-HRP (Southern Biotech, Birmingham, AL). Serum BAFF was measured by ELISA (RnD Systems, Minneapolis, MN). For assessment of B cell development, bone marrow was flushed from tibiae and femora of naïve mice and analysed by flow cytometry.

### Antibodies

The following antibodies were used for flow cytometry. Anti-CD4 (RM4.5) anti-CD25 (PC61), anti-CD44 (IM7), anti-CXCR5 (2G8), all BD. Anti-Foxp3 (FJK-16s), anti-PD-1 (J43) all Ebioscience. Anti-B220 (RA2-6B2), anti-CD23 (B3B4), anti-CD21 (7E9), anti-IgM (RMMM-1), anti-IgD (11-26c.2a), anti-CD138 (281–2) all Biolegend. Fixable viability stain 450 or propidium iodide (BD Biosciences) were used to exclude dead cells. Flow cytometry was performed on the Beckman Coulter Navios platforms and analysed using FlowJo software (TreeStar, Ashland, OR).

### Statistics

Data are presented with each dot representing a mouse and a bar representing median value. Prism 6 (Graphpad, San Diego, CA) software was used with analysis by Mann-Whitney U test. * p<0.05, **p< 0.01, ***p<0.001.

## Results

### Endogenous C3a does not exacerbate glomerular injury induced by passive transfer of anti-MPO IgG but promotes glomerular macrophage recruitment

**Fig 1:**
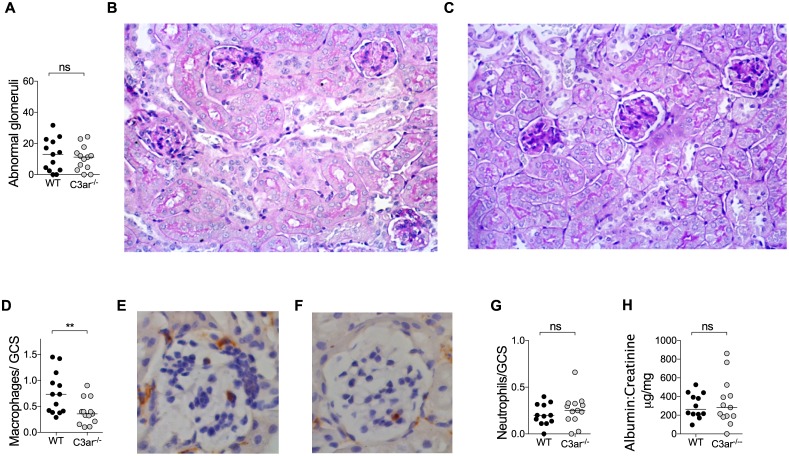
Endogenous C3a does not promote anti-MPO glomerulonephritis but supports macrophage recruitment to the inflamed glomerulus. Anti-MPO glomerulonephritis was induced in WT and C3ar^-/-^ mice (n = 14/group, data from two independent experiments) by injection of 100μg/g anti-MPO IgG and 0.5μg/g LPS. On day 7 histological glomerular injury was assessed. Both groups of mice developed glomerulonephritis including segmental necrosis. There was no difference in degree of injury (A) between WT (B) and C3ar^-/-^ (C) mice. Glomerular leucocyte influx was assessed by immunohistochemistry (n = 7/group). Compared to WT mice (D-E), the number of glomerular macrophages was reduced in C3ar^-/-^ mice. (F) There was no difference in glomerular neutrophils (G), Albuminuria, measured by urinary albumin: creatinine ratio was not different between groups (H). ** p<0.01 by Mann-Whitney U Test.

Passive transfer of anti-MPO IgG into mice induces a neutrophil mediated necrotising glomerulonephritis [25]. Glomerular injury is exacerbated by the co-administration of lipopolysaccharide (LPS) [26]. We used this enhanced model to investigate the role of the endogenous C3a in the effector phase of anti-MPO IgG induced glomerulonephritis. Wild type (WT) and *C3ar*^*-/-*^ mice received anti-MPO IgG and LPS. Renal injury was studied after 7 days. Both groups of mice developed glomerulonephritis with a similar degree of histological glomerular injury (Fig 1A–1C). However, fewer glomerular macrophages were observed in *C3ar*^*-/-*^ mice with glomerulonephritis (Fig 1D–1F). Numbers of glomerular neutrophils were not different between groups and, consistent with histological findings, albuminuria was similar between groups (Fig 1G and 1H). Therefore, in this model, the C3aR is not required for the development of anti-MPO IgG induced glomerular injury, but does promote macrophage infiltration to the inflamed glomerulus.

### The C3aR does not promote injury in experimental autoimmune anti-MPO glomerulonephritis

**Fig 2:**
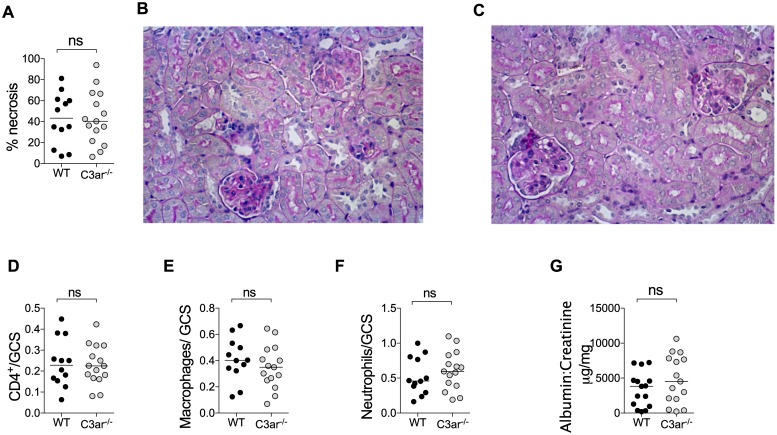
Endogenous C3a does not promote autoimmune anti-MPO glomerulonephritis. Autoimmunity to MPO was induced in WT and C3ar^-/-^ mice (n = 13-15/group, data from two independent experiments) by immunisation with 20μg MPO in FCA followed 7 days later by 10μg MPO in FIA. Disease was triggered on day 16 by i.v. injection of 0.12mg/g sheep anti-mouse GBM globulin in two divided doses and glomerular injury was assessed on day 21. Mice developed glomerulonephritis with focal areas of segmental necrosis, but there was no difference in the degree of injury (A) between WT (B) and C3ar^-/-^ (C) mice. Glomerular leucocytes were assessed by immunohistochemistry. The number of glomerular CD4^+^ cells (D), macrophages (E) and neutrophils (F) were similar between groups. Functional renal injury measured by urinary albumin: creatinine ratio was similar between groups (G).

We then examined the role of C3aR in an autoimmune model of anti-MPO glomerulonephritis that is mediated by the T cell effector response to glomerular MPO[3]. In mice, autoimmunity to MPO induced by immunisation does not result in ANCA of sufficient pathogenicity to cause disease. However, injection of a sub-nephritogenic dose of anti-glomerular basement membrane (GBM) globulin induces glomerular neutrophil recruitment and deposition of MPO. Glomerular MPO is recognised by antigen specific effector T cells with resulting necrotising glomerulonephritis[3,4]. Several strands of evidence confirm that this disease is due to MPO-specific T cell effectors. These include lack of injury in Mpo^-/-^ mice or OVA immunised mice, a similar degree of injury in B cell deficient mice and the induction of glomerulonephritis by transfer of MPO-specific, but not OVA-specific CD4+ T cell clones [3,4,27]. As this model is dependent on glomerular neutrophil recruitment by anti-GBM globulin, we first confirmed that this parameter was not affected by the absence of the C3aR (150 minutes after anti-GBM IgG, WT 1.17±0.17 vs. C3ar^-/-^ 1.13±0.16; mean ± SEM, neutrophils/glomerular cross section). Having excluded this potential confounder, we induced autoimmune anti-MPO glomerulonephritis in WT and C3ar^-/-^ mice. Both groups had similar severity of histological injury and albuminuria, with no differences in the numbers of neutrophils, macrophages or CD4^+^ T cells between WT and C3ar^-/-^ mice (Fig 2A–2G).

### C3a suppresses humoral autoimmunity to MPO

**Fig 3:**
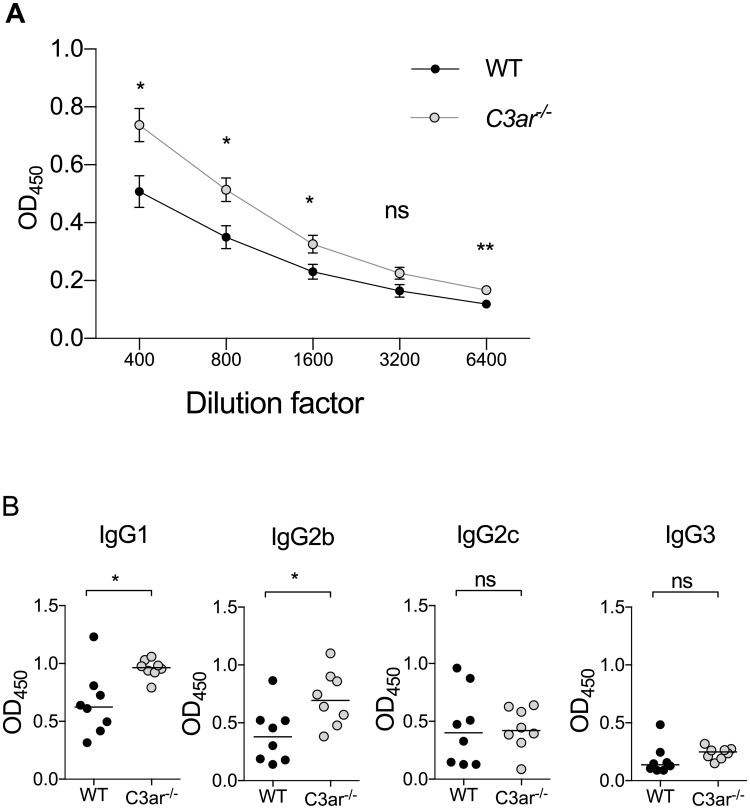
C3a suppresses anti-MPO humoral immunity. Total anti-MPO IgG titres and anti-MPO IgG subclass titres were measured by ELISA in WT and C3ar^-/-^ mice (n = 8/group). (A) Anti-MPO IgG titres were higher in C3ar^-/-^ mice compared to WT. Data shown as mean ± SEM. (B) This was due to elevated anti-MPO antibodies of the IgG1 and IgG2b subclasses. MPO-specific IgG2c and IgG3 were not different between groups. * p<0.05, ** p<0.01 by Mann-Whitney U Test.

As ANCA production is critical in the pathogenesis of AAV, we measured the development of anti-MPO humoral autoimmunity in this disease model. MPO-ANCA IgG titres were increased in C3ar^/-^ mice (Fig 3A and S2 Fig). Levels of anti-MPO IgG1 and IgG2b isotypes, but not IgG2c and IgG3 were significantly higher in C3ar^-/-^ than wild-type mice (Fig 3B).

**Fig 4:**
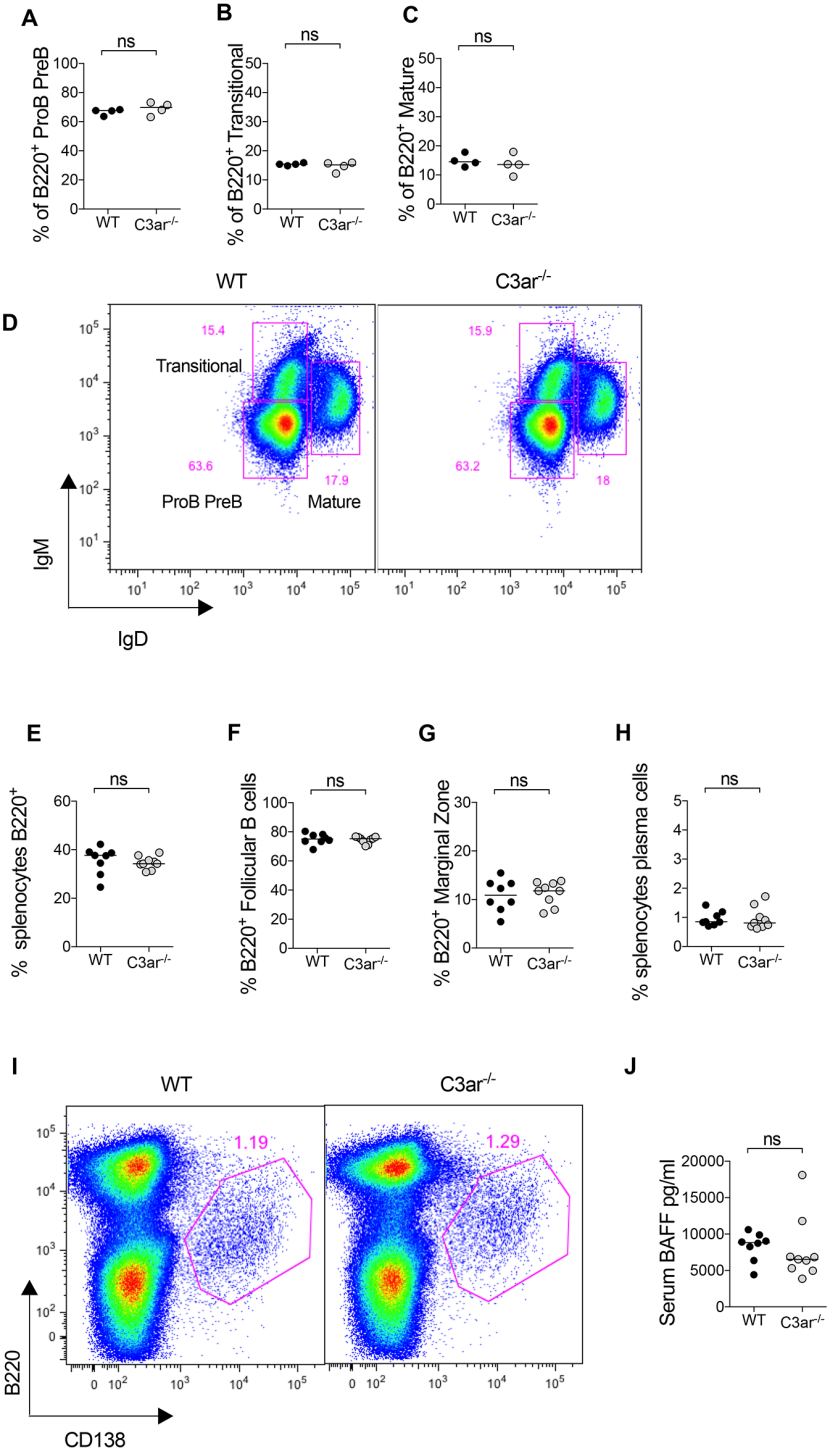
C3ar^-/-^ mice have normal B cell development in the spleen and bone marrow. Bone marrow cells were extracted from tibiae of naïve WT and C3ar^-/-^ mice (n = 4/group) and surface markers analysed by flow cytometry. (A-C) The proportion of B220^+^ cells that were preB or proB (IgM^low^IgD^low^), transitional (IgM^+^IGD^low^) or mature (IgM^+^IgD^+^) did not differ between groups. (D) Representative flow cytometry plot gated on B220^+^ cells in the bone marrow showing gating strategy. The splenic B cell compartment was analysed in WT and C3ar^-/-^ mice (n = 8/group) at the end of the autoimmune anti-MPO glomerulonephritis model. There was no difference in (E) the proportion of splenocytes that were B220^+^ B cells, (F) the proportion of B220^+^ cells that were CD21^+^CD23^+^ follicular B cells, or (G) CD21^+^CD23^low^ marginal zone B cells was similar between groups. (H) The proportion of splenocytes that were B220^low/int^CD138^+^ plasma cells also did not differ between groups. (I) Representative flow cytometry plot of splenocytes showing gating of plasma cells. (J) Serum B cell activating factor (BAFF) was similar between groups.

To further investigate the differences in humoral autoimmunity, we analysed B cell development in the bone marrow and spleen. In naïve mice, the proportion of bone marrow B cells that were proB-preB (B220^+^IgM^low^IgD^low^), transitional (B220^+^IgM^+^IgD^low^) or mature (B220^+^IgM^int^IgD^+^) was not different between WT and C3ar^-/-^ mice. In mice immunised with MPO in FCA, there was no difference in the total number of splenic B220^+^ B cells, Follicular B cells (B220^+^CD21^+^CD23^+^), marginal zone B cells (B220^+^CD21^+^CD23^low^) or plasma cells (B220^low/int^ CD138^+^) between groups (Fig 4). B cell activating factor (BAFF), expressed by myeloid and bone marrow stromal cells is elevated in AAV[28] and is important in B cell development and differentiation. Levels of serum BAFF in immunised WT and C3ar^-/-^ mice were not different between groups.

### C3a does not promote cellular autoimmunity to myeloperoxidase

**Fig 5:**
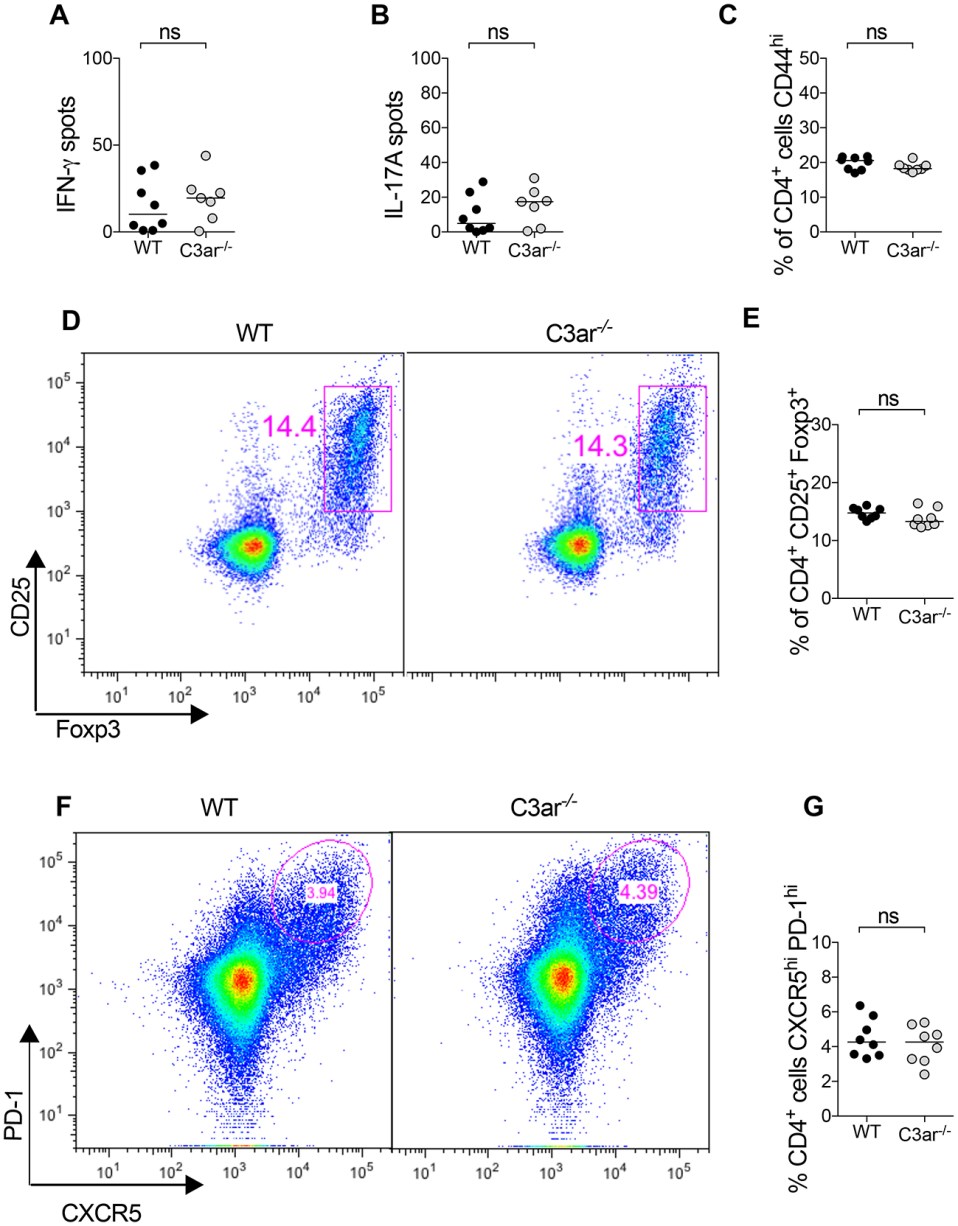
Endogenous C3a does not promote early cellular immunity to myeloperoxidase. To assess the early cellular immune response lymphocytes from the draining lymph nodes of WT and C3ar^-/-^ mice (n = 8/group) were studied 10 days after immunisation with 20 μg MPO in FCA. There was no difference between groups in Th1 and Th17 response measured by (A) IFN-γ and (B) IL-17A ELISPOT. (C) The proportion of activated CD44^+^CD4^+^ T cells was similar between groups. (D) Representative flow cytometry plots gated on CD4^+^ cells showing CD25^+^Foxp3^+^ Tregs. (E) The proportion of CD4 cells that were Tregs did not differ between groups. (F) Representative flow cytometry plots gated on CD4^+^ cells showing PD-1^hi^ CXCR5^hi^ T follicular helper cells. (G) There was no difference between groups in the proportion of CD4 T cells that had a TFH phenotype.

CD4^+^ T cells participate in AAV in several ways. They provide help to B cells for the generation of humoral immunity and also effect glomerular injury in crescentic glomerulonephritis via Th1 and Th17 responses [29–31]. *In vitro*, C3aR ligation on dendritic cells results in increased activation and functional capacity to activate T cells [32,33]. T cell intrinsic C3aR has also been reported to be important in Th1 responses and in inhibiting the generation of Foxp3^+^ T regulatory cells (Tregs) [34–36]. We therefore analysed cellular immune responses in mice at two time points: early (10 days) and later (21 days) after immunisation. Ten days after MPO immunisation there were no differences in MPO-stimulated Th1 or Th17 responses measured by IFN-γ and IL-17A ELISPOT (Fig 5A and 5B). Furthermore, proportions of CD4^+^ cells that were CD44^hi^ (activated T cells) (Fig 5C), or CD25^+^Foxp3^+^ Tregs (Fig 5D and 5E) were similar between groups. T follicular helper cells (TFH) are important for the formation of germinal centres and subsequent antibody response, the proportion of CD4 cells with CXCR5^hi^PD-1^hi^ TFH phenotype were also similar between groups (Fig 5F and 5G).

**Fig 6:**
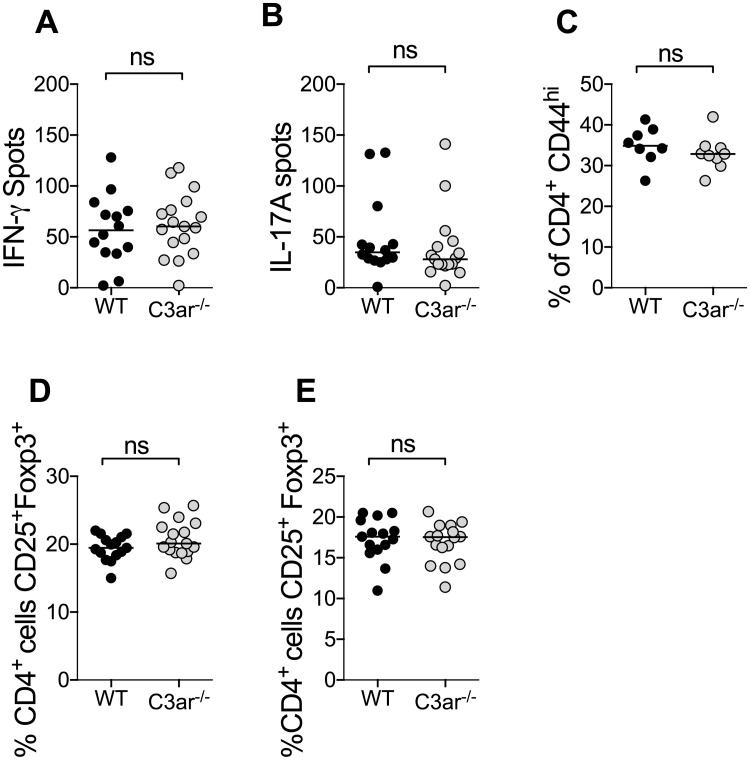
Endogenous C3a does not promote later cellular immunity to MPO in autoimmune anti-MPO glomerulonephritis. Cellular autoimmunity to MPO was assessed on day 21. Draining lymph nodes and spleen were removed from mice and a single cell suspension obtained. (A-B) There was no difference between groups in Th1 or TH17 response as measured by IFN-γ and IL-17A ELISPOT. (C) There was no difference between groups in the proportion of CD4 cells that had a CD44^+^ activated phenotype. There was also no difference in the proportion of CD4 cells that had a CD25^+^Foxp3^+^ regulatory phenotype in either the draining lymph nodes (D) or spleen (E).

Similar to findings at day 10, at day 21 there was no difference in MPO-stimulated IFN-γ and IL-17A producing cells measured by ELISPOT (Fig 6A and 6B). Additionally at this time point, the proportion of CD4 T cells that were CD44^+^ activated or CD25^+^Foxp3^+^ Tregs were similar, both in the draining lymph nodes and spleen(Fig 6C–6E).

## Discussion

A significant body of evidence implicates complement in the pathogenesis of AAV. To date, the C5aR has been identified as a key mediator of neutrophil activation and glomerular injury. Here, we assessed whether the signalling though the C3aR mediated autoimmunity or glomerular injury, and thus had potential as a therapeutic target. C3aR has previously been described to play both pathogenic and protective roles in experimental inflammatory diseases. Animal models in which the C3aR is pathogenic include antibody-induced arthritis[37], adriamycin induced nephropathy[38], complement mediated tubulointerstitial injury[21] and renal ischemia-reperfusion injury[39]. However, the C3aR is protective in lupus-like disease in the MRL/lpr mouse [40], endotoxic shock [22] and intestinal ischemia-reperfusion injury [18]. In the current studies, although we found that the C3aR had biological effects, there were no net effects on glomerulonephritis and renal injury in the two complementary disease models studied.

Passive transfer of anti-MPO IgG with LPS models pathological neutrophil activation with glomerular injury being caused by neutrophil degranulation, and the consequent release of inflammatory mediators and reactive oxygen species [25]. Whilst absence of the C3aR did not influence overall injury it was associated with an attenuation of glomerular macrophage accumulation, suggesting a role in this context for local C3a. *In vitro* and *in vivo* evidence supports a role for the C3aR in renal macrophage recruitment. *In vitro*, C3a acts as a chemotaxin in a murine macrophage cell line [41]. *In vivo* evidence derives from a model of complement induced renal injury in which *Crry*^*-/-*^*C3*^*-/-*^ kidneys are transplanted into syngeneic complement sufficient recipients, resulting in unrestricted renal complement activation with a inflammatory cell influx. When *Crry*^*-/-*^*C3*^*-/-*^ kidneys were transplanted into C3a*r*^*-/-*^ recipients, there was a marked attenuation of the monocyte-macrophage influx, suggesting a role for the C3aR in monocyte trafficking to areas of complement activation[21].

Whilst AAV is classically described to be “pauci-immune” with minimal glomerular complement deposition, detailed analysis of kidney biopsies from humans with AAV reveals evidence of local complement activation with detectable C3c, C3d and C5b-9 deposition[14,42,43], additional evidence for renal complement activation in AAV is the elevated urinary C3a observed in patients with active disease[42]. This complement activation, which is likely to be initiated by neutrophil secreted factors such as properdin [44], MPO [45] and neutrophil extracellular traps [46] may be one of the early signals contributing to macrophage recruitment to the inflamed glomerulus. Depletion studies have implicated macrophages as important injurious mediators in murine models of glomerulonephritis including disease induced by anti-MPO IgG[47,48]. In the current experiments, the observed reduction in glomerular macrophages in C3ar^-/-^ mice was not paralleled by reduced glomerular injury. This may be because when compared to studies using pharmacological or genetic depletion, the magnitude of the reduction in infiltrating macrophages was insufficient affect a change in injury.

While passive transfer of anti-MPO IgG allows study of the role of effector pathways responding to autoantibodies, it cannot be used to examine the contribution of the adaptive immune system to glomerular injury. AAV is an autoimmune disease in which both B and T cells play essential roles in the pathogenesis. We used the model of experimental autoimmune anti-MPO glomerulonephritis to model these processes and found that absence of C3aR did not affect albuminuria, histological injury or glomerular leucocyte influx. The discrepancy between results for glomerular macrophage infiltration between passive transfer of anti-MPO IgG and autoimmune anti-MPO glomerulonephritis is likely to be because the dominant signals driving glomerular macrophage recruitment in the model of autoimmune anti-MPO GN are T helper cell derived costimulatory molecules, such as CD154-CD40 interactions [49] and cytokines, such as IL-17A [29] and IFN-γ [50].

C3ar^-/-^ mice had an increased humoral immune response to MPO with higher MPO-ANCA titres. Elevated autoantibody titres associated with accelerated renal injury have also described in C3ar^-/-^ mice when backcrossed to the MRL lupus-prone strain [40]. In human cells, C3a has been reported to directly suppress polyclonal antibody response from isolated B lymphocytes, [20,51] whilst both activation and suppression of T lymphocytes have shown[34,51] C3ar^-/-^ mice have also been reported to have an enhanced Th2 response and higher anti-OVA IgG titres after epicutanteous sensitisation [52]. In contrast, models of infection have found that absence of the C3aR results in unaltered [53] or attenuated [54] humoral immunity. The enhanced humoral immunity in *C3ar*^*-/-*^ mice is not likely to be due to elevated C3 levels (and enhanced generation of C3d which is a potent B cell adjuvant), as serum C3 in this strain has previously be described to be similar to that of WT mice [37,40]. Because of the observed differences in anti-MPO IgG titres we investigated whether *C3ar*^*-/-*^ mice had any numerical or developmental alteration in the B lymphocyte compartment. However, B cell number and development in the spleen and bone marrow appeared similar to WT mice.

Given the previous descriptions of the important role that C3aR plays in influencing adaptive immunity we investigated the effect of absence of this receptor on the generation of T cell mediated immunity. We found no difference in the generation of Th1 or Th17 effector responses, or in the proportion of T cells that had a CD25^+^Foxp3^+^ regulatory phenotype. This is in contrast to previous reports of elevated T regulatory cells in *C3ar*^*-/-*^ mice[35,55]. The model of autoimmune anti-MPO glomerulonephritis relies on immunisation with MPO in Freund’s complete adjuvant to break immune tolerance and generate autoimmunity. Other investigators who have reported increased T regulatory cells in *C3ar*^*-/-*^ mice used animal models that either require no adjuvant or use incomplete Freund’s adjuvant. It is possible that differences in the additional immune signals provided by the adjuvant may account for this discrepancy. Additionally, whilst C3aR expression in the mouse has previously been reported to be extensive in both myeloid and lymphoid lineages, this has been called into questions by recent findings using a C3aR reporter mouse in which expression of C3aR was evident on macrophages and some dendritic cell subsets but not bone marrow neutrophils, B or T cells [56,57]. In contrast, neutrophil, macrophage and T cell expression have been reported in humans[34,58–61]. Potential species differences in C3aR distribution should therefore be considered when interpreting studies using murine models.

In summary, these data suggest a role for C3a in driving glomerular macrophage recruitment and suppressing humoral immunity in these pre-clinical models of AAV. However, the lack of attenuation of immune mediated glomerular injury in either model does not support the C3aR as a putative therapeutic target in this disease.

## Supporting information

S1 Fig Mouse genotyping. Primers C1 and A201+ yield a fragment of 726 bp, denoting WT allele. Primers C1 and NeoA yield a fragment of 537 bp denoting the C3ar^-/-^ allele.
(TIFF)

S2 Fig Anti-MPO IgG titres. Anti-MPO IgG titres were measured by ELISA in WT and C3ar^-/-^ mice (n = 5-7/group). Anti-MPO IgG titres were higher in C3ar^-/-^ mice compared to WT. Data shown as mean ± SEM.
(EPS)
