# Supplementary figures and images for: The C3aR promotes macrophage infiltration and regulates ANCA production but does not affect glomerular injury in experimental anti-myeloperoxidase glomerulonephritis
Source: PLoS One. 2018 Jan 9;13(1):e0190655. doi: 10.1371/journal.pone.0190655 (PMC5760037; doi:10.1371/journal.pone.0190655)

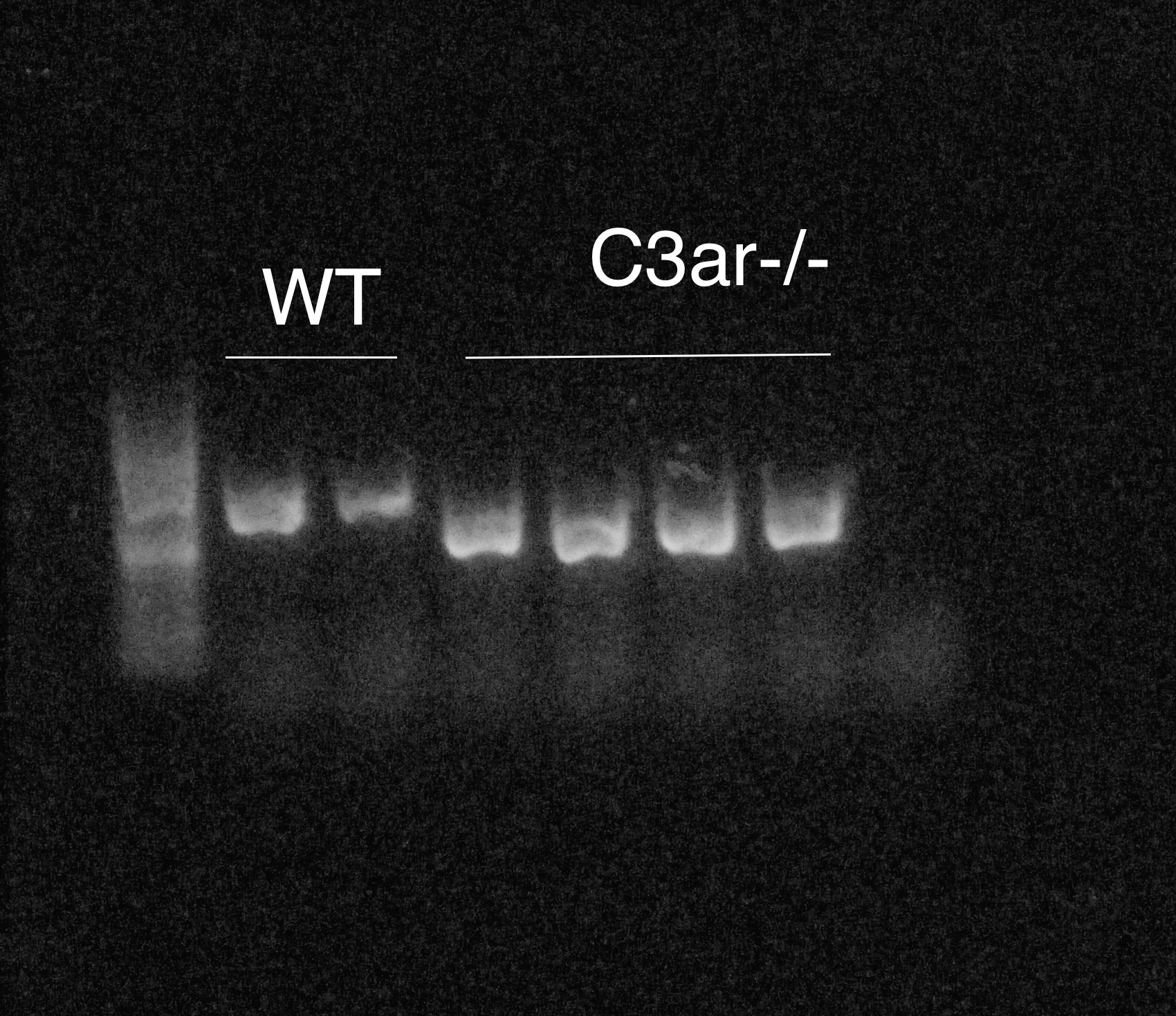

Supplement: S1 Fig — Primers C1 and A201+ yield a fragment of 726 bp, denoting WT allele. Primers C1 and NeoA yield a fragment of 537 bp denoting the C3ar-/- allele. (TIFF) [file pone.0190655.s001.tiff]
